# The microcirculation in the first days of ICU admission in critically ill COVID-19 patients is influenced by severity of disease

**DOI:** 10.1038/s41598-024-56245-5

**Published:** 2024-03-18

**Authors:** Fleur Brouwer, Can Ince, Jiska Pols, Zühre Uz, Matthias Peter Hilty, Mendi Sesmu Arbous

**Affiliations:** 1 Department of Intensive Care, Leiden University Medical Center, Leiden, The Netherlands; 2 Department of Intensive Care, Erasmus MC, University Medical Center, Rotterdam, The Netherlands; 3 Institute of Intensive Care Medicine, University Hospital of Zurich, Zurich, Switzerland

**Keywords:** Coronavirus disease 2019, Sublingual microcirculation, Cytocam, Hypoxia, Inflammation, Hypoxia, Imaging and sensing

## Abstract

The objective of this study was to investigate the relationship between sublingual microcirculatory parameters and the severity of the disease in critically ill coronavirus disease 2019 (COVID-19) patients in the initial period of Intensive Care Unit (ICU) admission in a phase of the COVID-19 pandemic where patients were being treated with anti-inflammatory medication. In total, 35 critically ill COVID-19 patients were included. Twenty-one critically ill COVID-19 patients with a Sequential Organ Failure Assessment (SOFA) score below or equal to 7 were compared to 14 critically ill COVID-19 patients with a SOFA score exceeding 7. All patients received dexamethasone and tocilizumab at ICU admission. Microcirculatory measurements were performed within the first five days of ICU admission, preferably as soon as possible after admission. An increase in diffusive capacity of the microcirculation (total vessel density, functional capillary density, capillary hematocrit) and increased perfusion of the tissues by red blood cells was found in the critically ill COVID-19 patients with a SOFA score of 7–9 compared to the critically ill COVID-19 patients with a SOFA score ≤ 7. No such effects were found in the convective component of the microcirculation. These effects occurred in the presence of administration of anti-inflammatory medication.

## Introduction

In December 2019, the outbreak of the Coronavirus (SARS-CoV-2) in Wuhan, China, induced a pandemic disease known as Coronavirus disease-2019 (COVID-19)^1,2^. The outbreak of the COVID-19 pandemic led to the need to treat a high number of patients with acute hypoxemic respiratory failure worldwide who were admitted to the Intensive Care Unit (ICU) for invasive mechanical ventilation due to moderate or severe acute respiratory distress syndrome (ARDS)-induced COVID-19 infection, hereafter referred to as COVID-19 patients^2,3^. Simultaneously, it posed challenges in understanding pathophysiology, clinical course and therapeutic resolution. A COVID-19 infection is associated with inflammatory and coagulation activation leading to death in 24% of the cases^4^.

Since 2019, we have experienced several COVID-19 waves and more knowledge has been gained on the pathophysiology. Endothelial cell dysfunction has been suggested to play a central role in the pathogenesis of COVID-19^5,6^. This has led to the hypothesis that endothelial cell injury may cause microcirculatory dysfunction. The microcirculation is therefore identified as a target, by direct observation, to better understand the pathogenesis of COVID-19^7^.

Sublingual microcirculation can be monitored with a hand-held vital microscope (HVM) based on incident dark-field (IDF) imaging^8^. The development of an automated quantitative microcirculatory analysis platform, called MicroTools^9,10^, provides an unique tool for the quantitative evaluation of microcirculatory alterations in COVID-19 patients.

A few studies have investigated the sublingual microcirculation in patients with a COVID-19 infection^11–16^. Recently, a multicenter study of the sublingual microcirculation in mechanically ventilated COVID-19 patients was carried out to assess how precisely the COVID-19-associated inflammatory reaction can lead to microcirculatory dysfunction^11^. These measurements in COVID-19 patients showed a decrease in the diffusion distances between the capillaries in parallel with an increase in capillary hematocrit (cHct) and capillary-to-systemic hematocrit ratio (cHct/sHct ratio) as a response to hypoxemia caused by respiratory failure suggesting a microcirculatory compensatory mechanism that is able to increase its oxygen extraction capacity by increasing red blood cell (RBC) availability. This microcirculatory compensatory mechanism for COVID-19-induced hypoxemia is also described in other studies^12,15^. Nevertheless, this microcirculatory compensatory mechanism was affected when the Sequential Organ Failure Assessment (SOFA) score was higher than a threshold of 10^11^. This may indicate that the microcirculatory compensation mechanism is influenced by the severity of illness^11^.

However, a limitation of these studies is the heterogeneity regarding the timing of the microcirculatory measurement relative to the time of ICU admission. In addition, the standard therapy for a COVID-19 infection has been changed since the beginning of the COVID-19 pandemic. Currently, all COVID-19 patients receive corticosteroids (i.e. dexamethasone and an interleukin (IL)-6 inhibitor tocilizumab) at ICU admission, targeting the inflammatory response and thus probably the microcirculation^17,18^. Indeed, early clinical and experimental studies have shown that corticosteroids have a beneficial effect on microcirculatory alterations in sepsis^19,20^.

Therefore, this study aimed to assess sublingual microcirculatory parameters of critically ill COVID-19 patients according to the severity of the disease, expressed by the SOFA score, in the initial period of ICU admission, during a phase of the COVID-19 pandemic with corticosteroids and an IL-6 inhibitor as standard treatment. We hypothesized that there is a relationship between the SOFA score and the diffusive aspects of the microcirculation.

## Results

**Figure 1:**
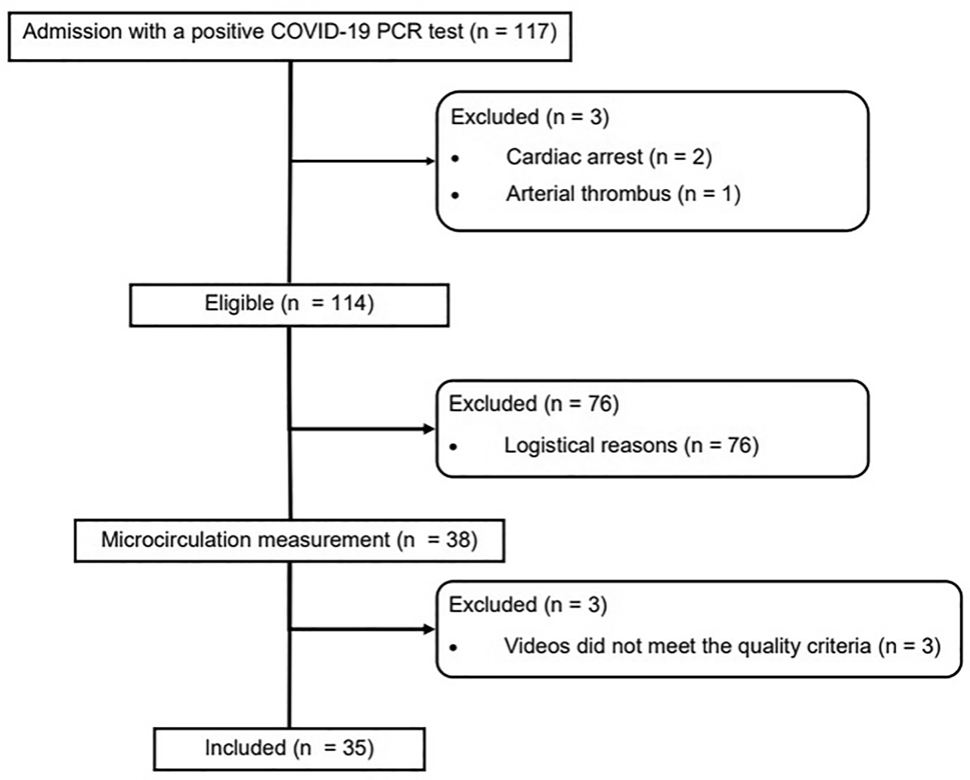
Flowchart. COVID-19: coronavirus disease-2019; PCR: polymerase chain reaction.

Sublingual microcirculation measurements were performed in 35 COVID-19 patients within the first five days of ICU admission (Fig. 1). Because all the patients were admitted to the ICU at the beginning of 2021, only four patients (11.4%) had received their first vaccination. Three patients were excluded from further analysis because all videos did not meet the quality criteria.

### Clinical demographics

The demographic characteristics, comorbidities, COVID-19 clinical management and clinical outcomes of the patients can be found in Additional file 1. The median age of the COVID-19 patients was 66 years (IQR: 53–70) and most of the patients were male (71.4%). All patients had a SOFA score < 10 in the first 24 h of ICU admission. The most common comorbidities were arterial hypertension (40%), diabetes (37.1%) and obesity (48.6%) with a mean BMI value of 30.0 ± 5.3 kg/m^2^.

Thirty-three patients (94.3%) were mechanically ventilated and two patients (5.7%) received nasal high-flow therapy at the time of the microcirculation measurement. Fourteen patients were transferred (40.0%) from another hospital. All patients received dexamethasone (6 mg per day, for 10 days) and tocilizumab (600 mg intravenous, once) at ICU admission in the LUMC or at the ICU of the hospital at which patients were admitted before they were transferred to the LUMC. The median time of ICU admission to the microcirculation measurement was 1 day (IQR: 0.0–2.0). On average, COVID-19 symptoms started 11.6 ± 4.8 days before the microcirculation measurement. Twelve patients (34.3%) died in the hospital, of which eleven patients (31.4%) were in the ICU. One patient (2.9%) received VV-ECMO and nine patients (25.7%) acquired a pulmonary embolism during their ICU stay. Furthermore, thirteen patients (37.1%) received methylprednisolone during their ICU stay due to persistent respiratory deterioration and no response to prone position.

A median SOFA score of 7 was taken as a cutoff point to divide the patient into low (SOFA ≤ 7) and high (SOFA > 7) SOFA score groups. COVID-19 patients with a SOFA score > 7–9 were older (68.5 (63.8–74.5) years vs. 56.0 (51.0–68.5); P = 0.018), received more often methylprednisolone (57.1% vs. 23.8%; P = 0.046) and had a longer length of mechanical ventilation (17.0 (7.0–29.5) days vs. 7.0 (5.0–15.0) days; P = 0.046).

### Macrocirculatory hemodynamics

All clinical, laboratory and treatment variables, at the time of the microcirculation management, can be found in Additional file 2. COVID-19 patients were considered hypoxemic, as indicated by their partial pressure of oxygen (PaO_2_) levels (9.7 kPa, IQR: 8.8–10.6) and PaO_2_/FiO_2_ (PF)-ratio (19.7 ± 5.8). PaO_2_ levels and PF-ratios did not significantly differ between the low and high SOFA group. Twenty-five patients (71.4%) received noradrenalin (with a maximum dose of 0.41 mg/kg/min) and twenty-six patients (74.3%) received heparin. There were no significant clinical, laboratory and treatment differences between the low and high SOFA score group, except for the total fluid balance and the cumulative fluid balance. The high SOFA score group showed a significantly higher total fluid balance (1229.3 ± 797.9 mL vs. 540.7 ± 820.5 mL; P = 0.019) and a higher cumulative fluid balance (2162.3 ± 1455.4 mL vs. 456.9 ± 2463.8 mL; P = 0.026).

### Microcirculatory hemodynamics

**Figure 2:**
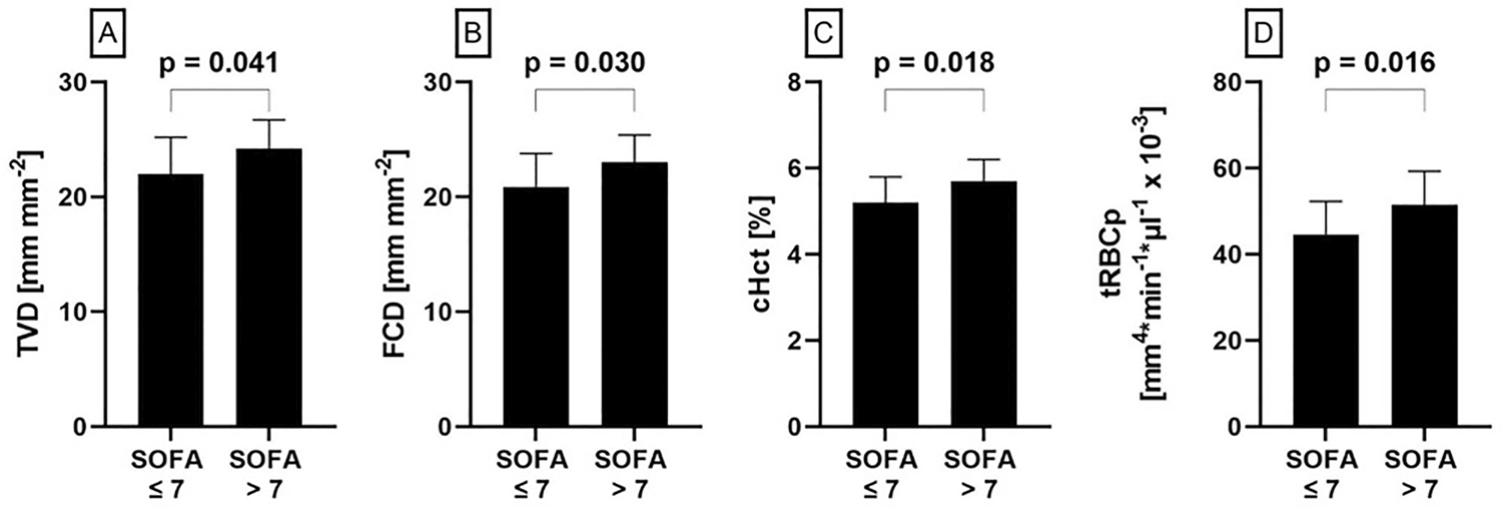
The critically ill coronavirus disease 2019 (COVID-19) patients in the high Sequential Organ Failure Assessment (SOFA) score group had a higher tissue vessel density (TVD) (**A**), a higher functional capillary density (FCD) (**B**), a higher capillary hematorcit (cHct) (**C**) and higher tissue red blood cell perfusion (tRBCp) (**D**) compared to the patients in the low SOFA score group.

Critically ill COVID-19 patients in the high SOFA score group showed a higher TVD (24.2 ± 2.5 vs. 22.0 ± 3.2; P = 0.041) and FCD (23.0 ± 2.4 vs. 20.9 ± 2.9; P = 0.030) than the critically ill COVID-19 patients in the low SOFA score group (Fig. 2A, B and Table 1). However, the PPV in the high and low SOFA score group was similarly close to 100% (Table 1). Moreover, despite a compatible systemic hematocrit (sHct) (0.38 ± 0.03 vs. 0.39 ± 0.05; P = 0.610), cHct was higher in the high SOFA score group (5.7 ± 0.5 vs. 5.2 ± 0.6; P = 0.018) (Fig. 2C and Table 1). This results in an almost significantly higher cHct/sHct ratio in the high SOFA score group indicating a shift of RBCs from the systemic circulation to the microcirculation (Table 1). Furthermore, the high SOFA score group had significantly higher tRBCp (51.5 ± 7.8 vs. 44.6 ± 7.7; P = 0.016) (Fig. 2D and Table 1).

**Table 1:** Microcirculatory parameters in critically ill COVID-19 patients are dependent on the severity of the disease.

	COVID-19 patients [N = 35]	SOFA ≤ 7 [N = 21]	SOFA > 7–9 [N = 14]	p-level
TVD [mm mm^−2^]	22.9 ± 3.1	22.0 ± 3.2	24.2 ± 2.5	**0.041**
FCD [mm mm^−2^]	21.7 ± 2.8	20.9 ± 2.9	23.0 ± 2.4	**0.030**
PPV [%]	95.0 ± 2.7	94.9 ± 3.1	95.0 ± 2.0	0.959
RBCv [µm s^−1^]	321.2 [303.2–337.5]	321.2 [301.3–338.3]	323.5 [304.3–334.2]	0.934
cHct [%]	5.4 ± 0.6	5.2 ± 0.6	5.7 ± 0.5	**0.018**
cHct/sHct ratio [l]	0.14 ± 0.02	0.13 ± 0.03	0.15 ± 0.02	0.078
tRBCp [mm^4^ * min^−1^ * µl^−1^ × 10^–3^]	47.4 ± 8.4	44.6 ± 7.7	51.5 ± 7.8	**0.016**

COVID-19: coronavirus disease-2019; cHct: capillary hematocrit, cHct/sHct ratio: capillary-to-systemic hematocrit ratio; FCD: functional capillary density; PPV: proportion of perfused vessels; RBCv: red blood cell velocity; SOFA: Sequential Organ Failure Assessment; tRBCp: tissue red blood cell perfusion; TVD: total vessel density.

Significant values are in bold.

## Discussion

The main finding of this study was that COVID-19 patients with a SOFA score > 7–9 showed higher values for the diffusive microcirculatory parameters (TVD, FCD and cHct) than patients with a SOFA score ≤ 7 in the initial period of ICU admission (median time between ICU admission and the microcirculation measurement was one day). No such effects were found in the convective component of microcirculatory tissue perfusion, i.e. RBCv. Furthermore, the new tRBCp parameter, combining the diffusive and convective component of microcirculatory tissue perfusion, was higher in the COVID-19 patients with a SOFA score > 7–9 indicating a better perfusion of the tissues by RBCs. Notably, these effects occurred in the presence of the administration of anti-inflammatory medication.

To contextualize these findings, it is essential to compare our results with two other notable investigations on critically ill COVID-19 patients and the microcirculation, namely the study by Favaron et.al.^11^ and Abou-Arab et.al.^13^.

Favaron et.al. proposed that the increase in TVC, FCD and cHct reflects a “microcirculatory compensatory response”, indicating the recruitment of the microcirculation to increase oxygen capacity in response to COVID-19-induced hypoxemia^11^. These effects were present in COVID-19 patients with a SOFA score < 10 but not in patients with a SOFA score ≥ 10. However, our study population consisted of critically ill COVID-19 patients with a SOFA score ranging from 5 to 9 and demonstrated that patients with a SOFA score > 7–9 had increased diffusive microcirculation compared to patients with a SOFA score ≤ 7. So from both studies, we still cannot make any statements as to the precise relation between the microcirculatory response and the severity of illness.

Abou-Arab et al. categorized patients into a severe group (i.e. having a respiratory rate ≥ 30/min of oxygen saturation of ≤ 90% on room air or signs of severe distress syndrome) and a critical group (i.e. having respiratory failure requiring mechanical ventilation of shock or organ failure that required ICU care)^13^. Their critical group, with a median SOFA score of 10 (IQR: 7–13), showed higher RBCv levels and higher vessel density, suggesting a “microcirculatory compensatory mechanism” compared to the severe group, with a median SOFA score of 3 (IQR: 1–4). In contrast, our study involved patients with a SOFA score < 10 (7 days, IQR: 6–8) and a significant proportion of our patients were mechanically ventilated during the microcirculation measurements (94.3%), presenting a distinctive clinical context. Our results differ from Abou-Arab et.al. emphasizing the complexity of microcirculatory responses at different severity levels of COVID-19.

Combining our results and those of Favaron et.al. and Abou-Arab et.al. makes it challenging to precisely explain the behavior of the diffusive aspects of the microcirculation in COVID-19 patients. The pattern evolving from Favaron’s findings suggests an increase in the diffusive components in patients with a SOFA score < 10, which is lost in patients with a SOFA score ≥ 10. Although our study did not directly compare to healthy volunteers and did not include patients with a SOFA score ≥ 10, we observed an increase in diffusive parameters in patients with SOFA score > 7–9, which is still below the higher SOFA score group of Favaron et.al. (SOFA score ≥ 10), compared to patients with a SOFA score ≤ 7. As the SOFA ranges of the study populations in the Favaron and our study were different, it is thus far not possible to conclude or even speculate about patterns in the diffusive adaptation of the microcirculation.

Besides the variation in the definition of critical illness, the timing of the microcirculation assessments represents a second factor contributing to the divergent outcomes. Abou-Arab et.al. conducted measurements within 24 hours after ICU admission, capturing the early disease state of COVID-19 patients^13^. This aligns with our study, as the median time of measurement after ICU admission in our study population was 1 day (IQR: 0–2). In contrast, Favaron et.al. measured the microcirculation at a later time point, with a median of 7 days (IQR: 3–12) after ICU admission^11^, potentially providing insight into the evolving nature of microcirculatory changes over time.

The third factor contributing to the divergent results is the choice of analysis method. Abou-Arab et.al. utilized AVA (Automated Vascular Analysis 3.2, Microvision Medical, Amsterdam, the Netherlands), a semi-automated software with manual vessel segmentation, while Favaron et.al. and our study applied Microtools, an automated full-frame analysis software. However, our approach placed a specific emphasis on the diffusive and convective components of the microcirculation, incorporating the newly introduced tRBCp parameter. Methodological distinctions in quantifying microcirculatory parameters across the studies could also have contributed to variations in the observed outcomes, emphasizing the significance of standardized analysis methods.

The observed increase in diffusive parameters (TVD, FCD and cHct) and tRBCp levels in the patients with a SOFA score > 7–9 (compared to patients with a SOFA score ≤ 7), despite no differences in respiratory parameters, like PF-ratios and PaO_2_ levels between the two SOFA groups (Additional file 2), prompts an evaluation of the interpretation of microcirculatory recruitment in the sublingual area. This becomes especially relevant given that a substantial proportion of the SOFA increase in this population is linked to the respiratory component. The whole study population had a homogeneously low PF-ratio (19.7 ± 5.8) and low PaO_2_ levels (9.7 kPa, IQR:8.8–10.6). We, therefore, could not link the microcirculatory differences in the two SOFA groups to the standard respiratory parameters as measured in the two SOFA groups. The concept of a “microcirculatory compensatory response” proposed by Favaron et.al.^11^, although occurring in a hypoxic context, may be linked to the mechanism of microcirculatory adaptation observed in healthy mountaineers exposed to hypoxia at high altitudes^21^. The term “happy hypoxia” describes the remarkable ability of COVID-19 patients to cope with low PaO_2_ levels similar to quite low PaO_2_ levels at high altitudes^22^. COVID-19 patients may tolerate such low PaO_2_ levels through a “microcirculatory compensatory response”, achieved by reducing the diffusion distances between the capillaries (increased TVD and FCD) and shifting RBCs from the systemic circulation to the microcirculation (increased cHct) to increase their oxygen extraction capacity. Our results, however, could not confirm this.

We searched for a pathophysiologic explanation for the increased diffusive microcirculatory parameters in the patients with a SOFA score > 7–9 compared to those with a SOFA score ≤ 7. This was challenging because we also observed that these patients had a higher positive fluid balance. Therefore, one would expect lower values for the diffusive parameters due to tissue edema, leading to increased distances on the microcirculatory level^23–25^. Upon examining the available evidence to elucidate this phenomenon, our conclusion is limited to the speculation that in our study population hypoxia appears to be the primary stimulus. However, the interrelationship between various stimuli influencing the diffusive capacity of the microcirculation seems to be very complex. With the existing evidence, we are unable to provide a more definitive explanation.

Much more research is needed to clarify the mechanisms of microcirculatory response to hypoxia in different subpopulations, e.g. healthy mountaineers and COVID-19 patients. While our study showed differences in diffusive microcirculatory parameters in two groups with different SOFA scores, an important factor contributing to the generalizability should be emphasized and this is the fact that all patients received tocilizumab and dexamethasone which will have influenced capillary leakage due to the effect of the inflammatory medication on the vascular barrier function. Thus our results can only be extrapolated to a specific subset of critically ill patients, namely with a SOFA score ≤ 9 and all having received dexamethasone and tocilizumab. Furthermore, the difference in cHct cannot be adequately explained, as all patients in our cohort were treated with tocilizumab and dexamethasone. This suggests that further research is necessary to better understand the underlying mechanisms. It is possible that the use of these medications may influence endothelial function, and additional studies are needed to investigate the specific impact of these treatments on the vascular system.

Firstly, it is crucial to emphasize that the absence of a control group makes it challenging to attribute microcirculatory changes to hypoxia. An appropriate control group could have been a group of healthy volunteers or patients with mild or severe ARDS without a COVID-19 infection. Moreover, the absence of a power calculation or sample size estimation raises concerns about the potentially small size of the study population. However, there was almost no knowledge available regarding the microcirculatory response to COVID-19 at the time of our study, on which we could have based a formal sample size calculation. Moreover, during the overwhelming pandemic, we were focused on acquiring scientific knowledge as quickly as possible, to be able to understand COVID-19 better and be able to better treat our COVID-19 patients. Another limitation is the heterogeneity in the timing of measurements, capturing COVID-19 patients at various stages of their disease. Despite the median from ICU admission to microcirculation measurement being relatively short at just 1 day (IQR: 0–2), challenges in logistics, transfers between hospitals, and the overwhelming presence of COVID-19 patients made it difficult to perform time-standardized measurements. Furthermore, this study did not specifically focus on leukocytes and microcirculatory RBC aggregates, which could provide more insights into COVID-19-induced hyperinflammation and hypercoagulation. Lastly, it must be kept in mind that sublingual microcirculatory alterations do not reflect lung microcirculation. The lung microcirculation could have been more compromised than the sublingual microcirculation resulting in a reduced oxygen-extraction capacity of the lung causing hypoxemia.

## Conclusion

In our study of a COVID-19 population receiving anti-inflammatory therapy, we found an increase in diffusive capacity (TVD, FCD and cHct) of the microcirculation and an increase in tRBCp in the patient with a SOFA score > 7–9 compared to patients with a SOFA score ≤ 7. Despite both groups displaying comparable systemic hypoxemia, as indicated by similar PaO_2_ and FiO_2_ levels, the observed increase in TVD and FCD raises questions about the specific microcirculatory response to COVID-19-induced hypoxemia. Comparisons with other studies highlighted the complexity of the microcirculatory response in COVID-19, with divergent outcomes linked to variations in critical illness definitions, timing of microcirculation measurements, and methodological discrepancies. The observed increase in diffusive parameters, even in patients with SOFA scores below the higher range in other studies, emphasized the nuanced nature of microcirculatory responses in COVID-19. While this increase in TVD and FCD might be similar to that which occurs in healthy individuals during exposure to hypoxia, the exact microcirculatory response to COVID-19-induced hypoxemia should be further explored in future studies.

## Methods

### Ethics approval

This single-center observational cross-sectional study was conducted in the ICU at the Leiden University Medical Center (LUMC), Leiden, The Netherlands. The study protocol was approved by the LUMC Institutional Review Board for COVID-19 studies. For this study informed consent was obtained by an opt-out procedure, which was approved by the LUMC Institutional Review Board for COVID-19 studies, protocol number CoCo 2021-018. All patients received a letter upon admission to state that clinical data could be used for research purposes and that they could opt out if they would not give informed consent. None of the admitted patients declined consent. Research data were pseudonymized and securely stored, according to the General Data Protection Regulation. The study was conducted in accordance with the ethical standards laid down in the 1964 Declaration of Helsinki and its later amendments^26^.

### Inclusion of patients

Patients with a confirmed COVID-19 infection, based on a positive polymerase chain reaction (PCR) test result in combination with the presence of typical radiological findings according to COVID-19 Reporting and Data System (CO-RADS), who were admitted to the ICU of the LUMC from March 2021 to June 2021 were included in this study. Patients could be admitted to the ICU via the emergency department, the hospital ward or as a transfer from another hospital from the emergency room of the ICU. Exclusion criteria were age < 18 years and having maxillofacial trauma or known tumor(s) in the mouth or throat area. All patients were treated according to prevailing COVID-19 treatment modalities: dexamethasone (6 mg per day, for 10 days) and tocilizumab (600 mg intravenous, < 24 h after ICU admission, once) at ICU admission in the LUMC (or at the ICU of the hospital at which patients were admitted before they were transferred to the LUMC). Methylprednisolone (1000 mg per day, for 3 days) was started (after 10 days of dexamethasone) by persistent respiratory deterioration and no response to prone position to mitigate the cytokine storm and inflammatory response.

In this phase of the pandemic, every ICU patient received a high prophylactic dose of heparin. This prophylactic dose was doubled compared to the regular prophylactic dose but was still classified as prophylactic heparin. In cases where pulmonary embolism was confirmed, patients were administered therapeutic intravenous heparin. Fluid and vasopressor therapy was delivered according to standard clinical practice for clinically ill patients.

### Data collection

For each patient, demographic data from the hospital’s electronic patient dossier (EPD) system was collected at ICU admission. Recorded data included age, sex, body mass index (BMI), Acute Physiology and Chronic Health Evaluation (APACHE) IV score, SOFA score, being transferred from another hospital, time between the start of COVID-19 symptoms and microcirculation measurement and time between ICU admission and microcirculation measurement. Clinical outcome data were extracted from the EPD system in the three-month follow-up time and include duration of mechanical ventilation, pulmonary embolism during ICU stay, Venovenous Extracorporeal Membrane Oxygenation (VV-ECMO) during ICU stay, ICU length of stay, hospital length of stay, ICU mortality, hospital mortality and transfer to another hospital. At the time of the microcirculation measurement, hemodynamic, respiratory, laboratory and treatment data were collected. The total fluid balance, i.e. the daily balance of input and output at the time of the microcirculation measurement, and the cumulative fluid balance, i.e. the sum of the total fluid accumulation at the time of the microcirculation measurement, were also collected.

### Measurements of the microcirculation

Sublingual microcirculation measurements were performed using IDF imaging (Cytocam™, Braedius Medical, Huizen, The Netherlands). The Cytocam is a third-generation HVM that enables non-invasive real-time visualization of the sublingual microcirculation. Detailed information on the working mechanism of the IDF imaging technique can be found elsewhere^8^.

The microcirculation measurement was performed within the first five days of ICU admission, preferably as soon as possible after ICU admission. First, the sublingual area was carefully cleaned with a suction or a gauze swab. The probe of the HVM was covered with a non-sterile disposable cap perpendicular to the area of interest in the sublingual area. Then, three videos of at least 3–5 s were recorded from different sublingual areas to minimize heterogeneity in the microscopic field of view according to the International guidelines^27^. Image sequences that did not meet the image quality criteria, focusing on illumination, image duration, focus, vessel content, stability and the absence of pressure induced by the probe^27,28^, were excluded from further analysis.

### Analysis of the microcirculation

Analysis of the videos was performed with the MicroTools automatic software (Active Medical, Leiden, The Netherlands) on the full frame image sequences^10^. The following microcirculatory parameters were retrieved from this off-line analysis: total vessel density (TVD), functional capillary density (FCD), proportion of perfused vessels (PPV), red blood cell velocity (RBCv), cHct and tissue red blood cell perfusion (tRBCp)^29^. Only small microvessels (diameter < 20 µm) were considered for the calculation of these parameters. The microcirculatory parameters measure the convective and diffusive capacity of the microcirculation for oxygen transport for the tissues. Convection is quantified by flow parameters, such as the velocity of the red blood cells (RBCs) inside the capillaries of the microcirculation^27,29^. The diffusive capacity of the microcirculation is quantified by parameters, such as TVD, FCD and cHct^27,29^. The recently introduced tRBCp parameter defines the concept of tissue perfusion in relation to adequate oxygen availability^27,29,30^. This parameter combines the diffusive and convective components of tissue perfusion. A detailed description of the microcirculatory parameters can be found in Additional file 3.

### Statistics

The Shapiro–Wilk normality test was used to determine whether the data were normally distributed. Normally distributed data were presented as means with standard deviations and non-normally distributed data were presented as medians with interquartile range (IQR). Driven by the signal provided by Favaron et.al.^11^ which was that the microcirculation behaved differently in the more severe (SOFA ≥ 10) versus less severe (SOFA < 10) critically ill patients, we wanted to have a scientifically reasonable contrast in our population. In our population the maximum SOFA score, unlike the study by Favaron et.al. was 9, so we established subgroups using the median SOFA score at admission^31^. A median SOFA score of 7 was subsequently selected as the threshold to categorize patients into two groups: those with low SOFA scores (SOFA ≤ 7) and those with high SOFA scores (SOFA > 7–9). Differences between the SOFA-score groups were tested with the independent sample t-test for normally distributed continuous data and with the Mann–Whitney U test for non-normally distributed continuous data. Categorical data were analyzed using the Chi-squared test or Fisher’s exact test. A P-value of < 0.05 was considered statistically significant. Statistical analysis was performed using IBM SPSS software version 25.0 for Windows (IBM Corp. Released 2017. IBM SPSS Statistics for Windows, Version 25.0. Armonk, NY: IBM Corp).

### Supplementary Information

Supplementary Information 1.

Supplementary Information 2.

Supplementary Information 3.

## Data Availability

The dataset used and/or analyzed during the current study are available from the corresponding author on reasonable request.

## Abbreviations

APACHE: Acute Physiology and Chronic Health Evaluation
ARDS: Acute respiratory distress syndrome
BMI: Body mass index
cHct: Capillary hematocrit
cHct/sHct ratio: Capillary-to-systemic hematocrit ratio
CO-RADS: COVID-19 Reporting and Data System
COVID-19: Coronavirus disease 2019
EPD: Electronic patient dossier
FCD: Functional capillary density
HVM: Hand-held vital microscopes
ICU: Intensive Care Unit
IDF: Incident dark-field
IL: Interleukin
IQR: Interquartile range
LUMC: Leiden University Medical Center
PaO_2_: Partial pressure of oxygen
PCR: Polymerase chain reaction
PF-ratio: PaO_2_/FiO_2_ ratio
PPV: Proportion of perfused vessels
RBC: Red blood cell
RBCv: Red blood cell velocity
SARS-CoV-2: Coronavirus
sHct: Systemic hematocrit
SOFA: Sequential Organ Failure Assessment
tRBCp: Tissue red blood cell perfusion
TVD: Total vessel density
VV-ECMO: Venovenous Extracorporeal Membrane Oxygenation
