# Supplementary material for: The microcirculation in the first days of ICU admission in critically ill COVID-19 patients is influenced by severity of disease
Source: Sci Rep. 2024 Mar 18;14:6454. doi: 10.1038/s41598-024-56245-5 (PMC10948764; doi:10.1038/s41598-024-56245-5)
Supplement: Supplementary file 1 — Supplementary Information 1. [file 41598_2024_56245_MOESM1_ESM.docx]

**Additional file 1**

**Demographic characteristics, comorbidities, management and clinical outcomes of COVID-19 patients.**

|  | COVID-19 patients [N = 35] | SOFA ≤ 7  [N = 21] | SOFA > 7-9 [N = 14] | P-level |  |
| --- | --- | --- | --- | --- | --- |
| Characteristics | | | | | |
| Age [years] | 66.0 [53.0 – 70.0] | 56.0 [51.0 – 68.5] | 68.5 [63.8 – 74.5] | ***0.018*** |  |
| Male [n (%)] | 25 / 35 (71.4%) | 15 / 21 (71.4%) | 10 /14 (71.4%) | 1.000 |  |
| Height [cm] | 174.5 ± 10.7 | 177.8 ± 10.8 | 169.5 ± 8.6 | ***0.022*** |  |
| Weight [kg] | 90.0 [80.0 – 98.0] | 89.0 [80.0 – 101.5] | 90.0 [75.8 – 94.5] | 0.396 |  |
| BMI [kg/m^2^] | 30.0 ± 5.3 | 30.0 ± 5.9 | 30.1 ± 4.7 | 0.955 |  |
| APACHE IV | 69.7 ± 27.2 | 60.3 ± 23.7 | 83.7 ± 26.7 | ***0.010*** |  |
| SOFA  Respiratory  Cardiovascular  Liver  Renal  Coagulation  Neurologic | 7.0 [6.0 – 8.0]  4 [3 – 4]  3 [3 – 4]  0 [0 – 0]  0 [0 – 0]  0 [0 – 0]  0 [0 – 0] | 6.0 [5.0 – 7.0]  4 [3 – 4]  3 [1 – 3]  0 [0 – 0]  0 [0 – 0]  0 [0 – 0]  0 [0 – 0] | 8.0 [8.0-9.0]  4 [4 – 4]  4 [3 – 4]  0 [0 – 0]  0.5 [0 – 1.3]  0 [0 – 0]  0 [0 – 0] | ***< 0.001***  0.429  ***<0.001***  0.495  ***0.022***  0.829  1.000 |  |
| Duration of COVID-19 symptoms before inclusion [days] | 11.6 ± 4.8 | 11.5 ± 5.0 | 11.7 ± 4.7 | 0.889 |  |
| Days from ICU admission to microcirculation measurement [days] | 1.0 [0.0 – 2.0] | 0.0 [1.0 – 2.0] | 0.5 [0.0 – 2.0] | 0.907 |  |
| Transferred from another hospital [n (%)] | 14 / 35 (40.0%) | 9 / 21 (42.9%) | 5 / 14 (35.7%) | 0.673 |  |
| Comorbidities | | | | | |
| Arterial hypertension  [n (%)] | 14 / 35 (40.0%) | 7 / 21 (33.3%) | 7 / 14 (50%) | 0.324 |  |
| Chronic heart disease  [n (%)] | 6 / 35 (17.1%) | 1 / 21 (4.8%) | 5 / 14 (35.7%) | ***0.028*** |  |
| Chronic pulmonary disease [n (%)] | 1 / 35 (2.9%) | 1 / 21 (4.8%) | 0 / 14 (0%) | 1.000 |  |
| Chronic kidney disease  [n (%)] | 2 / 35 (5.7%) | 0 / 21 (0%) | 2 / 14 (14.3%) | 0.153 |  |
| Chronic liver disease  [n (%)] | 0 / 35 (0%) | 0 / 21 (0%) | 0 / 14 (0%) | - |  |
| COPD [n (%)] | 5 / 35 (14.3%) | 3 / 21 (14.3%) | 2 / 14 (14.3%) | 1.000 |  |
| Diabetes Mellitus [n (%)] | 13 / 35 (37.1%) | 7 / 21 (33.3%) | 6 / 14 (42.9%) | 0.568 |  |
| Immunodeficiency [n (%)] | 2 / 35 (5.7%) | 0 / 21 (0%) | 2 / 14 (14.3%) | 0.153 |  |
| Obesity [n (%)] | 17 / 35 (48.6%) | 9 / 21 (42.9%) | 8 / 14 (57.1%) | 0.407 |  |
| COVID-19 clinical management | | | | | |
| Dexamethasone [n (%)] | 37 / 37 (100%) | 21 / 21 (100%) | 14 / 14 (100%) | - |  |
| Tocilizumab [n (%)] | 37 / 37 (100%) | 21 / 21 (100%) | 14 / 14 (100%) | - |  |
| Methylprednisolone [n (%)] | 13 / 37 (37.1%) | 5 / 21 (23.8%) | 8 / 14 (57.1%) | ***0.046*** |  |
| Mechanical ventilation  [n (%)] | 33 / 35 (94.3%) | 19 / 21 (90.5%) | 14 / 14 (100%) | 0.506 |  |
| ICU outcome | | | | | |
| Duration of mechanical ventilation [days] | 10.0 [6.0 – 23.5] | 7.0 [5.0 – 15.0] | 17.0 [7.0 – 29.5] | ***0.046*** |  |
| Pulmonary embolism during ICU stay [n (%)] | 9 / 35 (25.7%) | 5 / 21 (23.8%) | 4 / 14 (28.6%) | 1.000 |  |
| VV-ECMO during ICU stay [n (%)] | 1 / 35 (2.9%) | 0 / 21 (0%) | 1 / 14 (7.1%) | 0.400 |  |
| Length of stay ICU [days] | 11.0 [7.0 – 24.0] | 9.0 [6.5 – 16.5] | 21.0 [6.8 – 34.0] | 0.096 |  |
| Length of stay hospital [days] | 17.0 [9.0 – 32.0] | 17.0 [9.0 – 21.5] | 22.5 [6.8 – 36.5] | 0.278 |  |
| ICU mortality [n (%)] | 11 / 35 (31.4%) | 4 / 21 (19.0%) | 7 / 14 (50.0%) | 0.073 |  |
| Hospital mortality [n (%)] | 12 / 35 (34.3%) | 5 / 21 (23.8%) | 7 / 14 (50.0%) | 0.153 |  |
| Transferred to another hospital [n (%)] | 3 / 35 (8.6%) | 2 / 21 (9.5%) | 1 / 14 (7.1%) | 1.000 |  |

*APACHE IV = Acute Physiology and Chronic Health Evaluation IV; BMI= Body Mass Index; COPD = Chronic Obstructive Pulmonary Disease; COVID-19= Coronavirus disease-2019; ICU = Intensive Care Unit; SOFA = Sequential Organ Failure Assessment; VV-ECMO = Venovenous Extracorporeal Membrane Oxygenation;*

*Data presented as median [IQR], mean ± SD or n(%)*
