# Supplementary material for: The microcirculation in the first days of ICU admission in critically ill COVID-19 patients is influenced by severity of disease
Source: Sci Rep. 2024 Mar 18;14:6454. doi: 10.1038/s41598-024-56245-5 (PMC10948764; doi:10.1038/s41598-024-56245-5)
Supplement: Supplementary file 2 — Supplementary Information 2. [file 41598_2024_56245_MOESM2_ESM.docx]

**Additional file 2**

**Clinical, laboratory and treatment variables at time of the microcirculation measurement at ICU.**

|  | COVID-19 patients [N = 35] | SOFA ≤ 7  [N = 21] | SOFA > 7-9 [N = 14] | P-level |
| --- | --- | --- | --- | --- |
| Temperature [°C] | 36.4 ± 0.8 | 36.5 ± 0.7 | 36.3 ± 1.0 | 0.520 |
| Respiratory parameters | | | | |
| SaO_2_ [%] | 94.0 [93.0 – 95.0] | 94.0 [93.0 – 95.0] | 95.0 [91.8 – 95.0] | 0.960 |
| PaO_2_ [kPa] | 9.7 [8.8 – 10.6] | 9.6 [8.9 – 10.6] | 10.0 [8.1 – 11.0] | 0.934 |
| FiO_2_ [%] | 50.0 [45.0 – 60.0] | 50.0 [42.5 – 60.0] | 55.0 [45.0 – 60.0] | 0.538 |
| PF-ratio [ratio] | 19.7 ± 5.8 | 19.9 ± 5.5 | 19.3 ± 6.4 | 0.763 |
| PEEP [cmH_2_O] | 14.0 [10.0-16.0] | 14.0 [10.0 – 16.0] | 14.0 [12.0 – 16.0] | 0.474 |
| Circulatory parameters | | | | |
| HR [bpm] | 69.1 ± 18.1 | 68.4 ± 18.1 | 70.3 ± 18.8 | 0.766 |
| MAP [mmHg] | 80.0 [73.0 – 87.0] | 80.0 [73.5 – 88.5] | 77.5 [70.0 – 86.3] | 0.454 |
| Mean CVP [mmHg] | 11.3 ± 3.7 | 10.7 ± 2.9 | 11.9 ± 4.4 | 0.471 |
| Total fluid balance [mL] | 816.1 ± 869.9 | 540.7 ± 820.5 | 1229.3 ± 797.9 | ***0.019*** |
| Corrected cumulative fluid balance [mL] | 1139.0 ± 2258.2 | 456.9 ± 2463.8 | 2162.3 ± 1455.4 | ***0.026*** |
| Norepinephrine dose [μg/kg/min]  25/35 patients (71.4%) | 0.03 [0.00 – 0.09] | 0.01 [0.00 – 0.06] | 0.05 [0.02 – 0.11] | 0.066 |
| Laboratory parameters | | | | |
| Hb [none] | 7.9 ± 0.9 | 8.1 ± 1.1 | 7.8 ± 0.6 | 0.262 |
| sHct [%] | 0.39 ± 0.04 | 0.39 ± 0.05 | 0.38 ± 0.03 | 0.610 |
| Systemic leukocytes [10^9^/L] | 9.9 [6.7 – 13.5] | 8.9 [ 6.2 – 11.7] | 10.8 [9.5 – 15.1] | 0.077 |
| Lymphocytes [none] | 0.7 ± 0.4 | 0.6 ± 0.3 | 0.8 ± 0.5 | 0.253 |
| D-dimer [mg/L] | 1730.5 [941.3 – 6522.3] | 1591.0 [616.8 – 5127.8] | 2308.0 [1212.0 – 10043.5] | 0.323 |
| Lactate [mmol/L] | 1.8 [1.5 – 2.2] | 1.7 [1.4 – 1.9] | 2.0 [1.6 – 2.2] | 0.154 |
| Creatinine [µmol/L] | 83.5 [57.8 – 103.3] | 70.5 [57.3 – 91.8] | 98.0 [58.5 – 145.3] | 0.112 |
| LDH [U/L] | 477.0 [391.0 – 781.0] | 579.0 [427.0 – 687.5] | 412.0 [382.8 – 841.8] | 0.934 |
| Ferritin [µg/L] | 1336.0 [617.0 – 2263.0] | 1489.0 [888.0 – 2764.0] | 1246.0 [530.0 – 1897.8] | 0.300 |
| CRP [mg/L] | 45.2 [11.4 – 114.6] | 45.5 [13.0 – 132.8] | 39.8 [ 10.6 – 82.3] | 0.583 |
| Adjunct therapy | | | | |
| Mean Heparin dose [IE/24h]  26/35 patients (74.3%) | 0.0 [0.0 – 17280.0] | 0.0 [0.0 – 21120.0] | 0.0 [0.0 – 16560.0] | 0.778 |

*COVID-19 = Coronavirus disease-2019; CRP = C-reactive protein; CVP = Central venous pressure; FiO_2_ = Fraction of inspired oxygen; Hb = Hemoglobin; HR = Heart rate; LDH = Lactate dehydrogenase; MAP = Mean arterial pressure; PaO_2_ = The partial pressure of oxygen in blood; PEEP = Positive end-expiratory pressure; PF-ratio = PaO_2_ / FiO_2_ ratio; SaO_2_ = Oxygen saturation; sHct = Systemic hematocrit; SOFA = Sequential Organ Failure Assessment;*

*Data presented as median [IQR] or mean ± SD*
