# Supplementary material for: The microcirculation in the first days of ICU admission in critically ill COVID-19 patients is influenced by severity of disease
Source: Sci Rep. 2024 Mar 18;14:6454. doi: 10.1038/s41598-024-56245-5 (PMC10948764; doi:10.1038/s41598-024-56245-5)
Supplement: Supplementary file 3 — Supplementary Information 3. [file 41598_2024_56245_MOESM3_ESM.docx]

**Additional file 3**

**Standard microcirculatory parameters and proposed novel parameters computed with MicroTools automatic software, as requested by the second consensus on the assessment of sublingual microcirculation in critically ill patients (27).**

| Parameter | Abbreviation | Unit | Description | Physiological context |
| --- | --- | --- | --- | --- |
| Tissue vessel density | TVD | mm mm^-2^ | Sum of the length of all capillaries containing red blood cells, divided by field of view | Determinant of microcirculatory diffusion capacity |
| Proportion of perfused vessels | PPV | % | Weighted mean (by capillary segment length) of the categorical per-vessel ‘nonperfused’ property, which describes a per-space-time diagram-ridge velocity frequency histogram area under the curve proportion threshold transgression, after artifact elimination | Aspect of the heterogeneity of capillary perfusion |
| Functional capillary density | FCD | mm mm^-2^ | Sum of the length of all capillaries containing moving red blood cells, divided by field of view | Density of perfused capillaries as a determinant of microcirculation diffusion capacity |
| Red blood cell velocity | RBCv | µm s^-1^ | Weighted mean (by capillary segment length) of the absolute red blood cell velocity in all capillary segments within the field of view | Quantitative blood flow velocity as a determinant of microcirculatory convection capacity |
| Capillary hematocrit | cHct | 1 | Weighted mean (by capillary segment length) of the whole blood volume to red blood cell volume ratio in all capillary segments within the field of view | Corresponds to the distance of red blood cells within the boundaries of the capillaries and represents a determinant of microcirculatory diffusion capacity |
| Tissue red blood cell perfusion | tRBCp | mm^4^*min^-1^*µl^-1^ x 10^-3^ | Weighted mean (by capillary segment length) of the product of the integral over time of the linear displacement of red blood cells, capillary segment whole blood volume and cHct, divided by field of view | Perfusion of the tissue with RBCs as the most representative measure of tissue perfusion in a clinical and physiological context |
